# Expression profile of circRNA in peripheral blood mononuclear cells of patients with rheumatoid arthritis

**DOI:** 10.1186/s12920-022-01225-9

**Published:** 2022-04-04

**Authors:** Huangxin Lu, Yifan Yang, Dong Kuang, Ping Liu, Junping Yang

**Affiliations:** 1 Jiangxi University of Chinese Medicine, Nanchang, 330006 Jiangxi People’s Republic of China; 2 Shandong University of Traditional Chinese Medicine, Jinan, 250355 Shandong People’s Republic of China; 3 Nanchang University, Nanchang, 330027 Jiangxi People’s Republic of China; 4 Affiliated Hospital of Jiangxi University of Traditional Chinese Medicine, Nanchang, 330006 Jiangxi People’s Republic of China

**Keywords:** Rheumatoid arthritis, Circular RNA, Biomarker, Microarray chips, Hsa_circRNA_101328

## Abstract

**Background:**

Circular RNAs (circRNAs) is a newly discovered non-coding RNA that can be used as biomarkers in clinical blood samples. This study aims to screen differentially expressed circular RNAs in PBMCs of patients with rheumatoid arthritis (RA) to determine new biomarkers for the diagnosis of RA.

**Methods:**

The differentially expressed circRNAs in peripheral blood mononuclear cells (PBMCs) of 4 RA patients and 4 healthy participants were screened and analyzed by gene microarray technology. We then validated some of the differentially expressed circRNAs in PBMCs of 20 RA patients, 10 systemic lupus erythematosus (SLE) patients and 20 healthy participants using reverse transcription-quantitative polymerase chain reaction amplification (RT-qPCR). Spearman correlation test was performed to analyze the correlation between differentially expressed circRNAs and clinical variables in RA patients. Receiver operating characteristic (ROC) curves were calculated to evaluate the diagnostic value of circRNAs.

**Results:**

Differential analysis obtained 149 circRNAs with significant up-regulated expression and 250 circRNAs with significant down-regulated expression, which predicted the miRNA targets and binding sites. Compared with SLE and health control group, hsa_circ_101328 was found to be a common gene with differential expression of RA. Besides, correlation analysis revealed significant correlation between hsa_circ_101328 and positive CRP. ROC curve analysis showed that hsa_circ_101328 has the potential of RA diagnosis.

**Conclusion:**

We identified some dysregulated circRNAs in PBMCs from RA patients, and hsa_circ_101328 may be a novel and effective biomarker for early diagnosis of RA.

## Background

Rheumatoid arthritis (RA) is a clinically common autoimmune disease, which is distributed all over the world. It usually appears in the elderly, mainly among female [1]. The clinical features of RA are primarily related to symmetrical facet joint pain, swelling, morning stiffness and systemic inflammation. Because of active incidence, the illness would destroy joints at the late stage, eventually causing joint deformities and function loss if not systematically treated. As it increases disability rate and induces serious complications, RA is also known as immortal cancer [2]. For now, the clinical diagnosis of RA is mainly based on patient serological markers. However, as the sensitivity and specificity of testing indexes vary from one another, and there also exists false negatives in early related indexes, misdiagnosis may occur accordingly. Therefore, the diagnosis of RA remains difficult. Clinically, the treatment plan of RA mainly includes a combination of non-steroidal anti-inflammatory drugs, anti-rheumatic drugs, hormones and biological agents. In fact, it cannot completely cure the disease but effectively bring the disease under control and relieve the symptoms. Therefore, seeking out an effective early diagnostic method can provide an important theoretical basis for the development of early intervention in RA.

Circular RNA (circRNA) is an endogenous non-coding RNA that lacks the typical 3'end polyA tail and 5'end cap structure, and has a covalently closed loop structure. circRNAs are numerous in types and numbers [3], and have specificities among tissues, diseases, and species [4, 5]. CircRNAs are more stable than linear RNAs[6]. Additionally, circRNA plays a critical role in regulating gene transcription, binding and translating protein as well [7, 8], which explains the reason why circRNA has more potential to act as new biomarkers for disease diagnosis than other types of RNA. Also, studies have shown that circRNAs can regulate various biological processes such as inflammation and immune response by acting as miRNA sponges, which is also significant to autoimmune diseases [9]. At present, more and more studies have confirmed that circRNAs are important biomolecules for understanding the pathogenesis of related diseases. circRNAs are widely involved in the occurrence and development of diseases, such as various cancers and heart and brain diseases. However, these studies mainly focus on discussing the circRNA expressions in plasma, whole blood and serum, whereas the research on circRNAs in peripheral blood mononuclear cells (PBMCs) is relatively scarce. This study aims to screen the differentially expressed circRNAs in the PBMCs of RA patients to identify new biomarkers for the diagnosis of RA.

## Methods

### Study subjects

A total of 50 participants were recruited in this study: 20 RA patients, 10 systemic lupus erythematosus (SLE) patients and 20 gender- and age-matched healthy controls. All patients with rheumatoid arthritis were newly diagnosed in the Department of Rheumatology and Immunology, Affiliated Hospital of Jiangxi University of Chinese Medicine from May 2020 to September 2020, and they all met the RA classification standard revised by American College Rheumatology (ACR) and the European League Against Rheumatism (EULAR) in 2010 [10]. Patients with pregnancy, severe infection, malignancies, diabetes and other autoimmune diseases, and those who took any type of nonsteroidal anti-inflammatory drugs, glucocorticoids and biological agents before participating in the trial were excluded. healthy controls (n = 20) without autoimmune or inflammatory diseases and who were also unrelated to patients with autoimmune diseases were randomly enrolled from Affiliated Hospital of Jiangxi University of Chinese Medicine from May 2020 to September 2020. As a disease control, 10 SLE patients clinically confirmed by the diagnostic criteria of SLE [11] after excluding RA, were also enrolled from the from Affiliated Hospital of Jiangxi University of Chinese Medicine during the same period. The experimental study was approved by the Ethics Committee of Affiliated Hospital of Jiangxi University of Chinese Medicine (JZFYKYLL2019001) and signed informed consent forms obtained from the patients. Laboratory data for all participants are presented in Table 1.

**Table 1:** Characteristics of the study populations

Characteristic	T (n = 20)	C (n = 20)	SLE(n = 10)
Age (year)	62.80 ± 5.99	60.35 ± 6.71	59.80 ± 5.03
Sex (female/male)	7/13	9/11	4/6
CRP (mg/l)	31.60 ± 32.44	NA	NA
RF (IU/ml)	250.22 ± 261.81	NA	NA
Anti-CCP (U/ml)	118.60 ± 93.54	NA	NA
ESR (mm/h)	49.20 ± 24.96	NA	NA
Thrombocytocrit (%)	0.28 ± 0.08 ^a^	0.16 ± 0.03	0.22 ± 0.06
Platelet count (10^9^ L)	293.60 ± 100.36^a^	199.50 ± 50.23	209.4 ± 43.69^b^
Lymphocyte count (10^9^ L)	1.86 ± 0.57	1.97 ± 0.49	1.97 ± 0.48
White cell count (10^9^ L)	7.81 ± 1.93^a^	6.37 ± 1.61	6.87 ± 1.90
Neutrophil count (10^9^ L)	5.21 ± 1.67	4.47 ± 1.04	4.45 ± 1.02
Lymphocyte percentage (%)	24.45 ± 7.59	28.40 ± 2.78	23.63 ± 3.89
Percentage of neutrophils (%)	63.37 ± 16.16	56.15 ± 4.57	57.43 ± 5.73

Values presented as mean ± SD

CRP, C-reactive protein; RF, rheumatic factor; anti-CCP, anti-cyclic citrullinated peptide antibodies; ESR, erythrocyte sedimentation rate; NA, not available

^a^*P* < 0.05 T compared to C; ^b^*P* < 0.05 T compared to SLE

### PBMCs preparation and total RNA extraction

2.0–4.0 ml fasting venous blood of study objects was collected in Ethylene Diamine Tetraacetic Acid (EDTA) anticoagulant tube in early morning and sent to the laboratory within 5 h. The PBMCs were isolated using Lymphocyte Separation Medium (Human) (Solarbio, Beijing, China), TRIzol® reagent (Invitrogen; Thermo Fisher Scientific, USA) was added to the PBMCs and stored at ‑80˚C. Total RNA extraction from PBMCs specimens was carried out according to the manufacturer's instructions. The quality and integrity of RNA were measured by using the NanoDrop ND-1000 spectrophotometer (Thermo Fisher Scientific, USA) and agarose gel electrophoresis with 1% mass fraction.

### Microarray hybridization

Rnase R (Epicentre, USA) was added to the qualified total RNA. Enriched circRNAs were amplified using Arraystar Super RNA Labeling Kit (Arraystar, USA) and then transcribed into fluorescent cRNAs. After the purification of the labeled cRNAs with RNeasy kit (USA Kay root), the concentration and specific activity of labeled ones were measured by using the NanoDrop ND-1000 spectrophotometer (Thermo Fisher Scientific, USA). Add 5 μl of 10 × blocking reagent and 1 μl of 25 × fragmentation buffer per 1 μg of labeled cRNA for lysis, heat at 60 °C for 30 min, and finally add 25 μl of 12 × hybridization buffer to dilute the labeled cRNAs. Additionally, 50 μl of hybridization solution was injected into the spacer glass slide, assembled on the Arraystar Human circRNA V2 (8 × 15 K) chip (Arraystar, USA), and incubated in an Agilent hybridization oven at 65 °C for 17 h. After washing the slides, scan the array with G2505C gene scanner (Agilent, USA). The sequencing process of circRNA chip was carried out according to the protocol of KangChen Bio-tech, Shanghai, China.

### CircRNA and miRNA interaction

Among all differentially expressed circRNAs detected by microarray technology, we selected the top 8 up-regulated and down-regulated circRNAs in PBMC for the next analysis. Arraystar miRNA target gene prediction software based on TargetScan and MiRanda is used to predict the miRNA that binds to abnormally expressed circRNAs.

### The validation of differentially expressed circRNAs

QRT-PCR was performed with PrimeScript™ RT kit (Takara Bio Inc.) and SYBR Premix Ex Taq™ II (Takara Bio Inc.), respectively. Quantitative reverse transcription polymerase chain reaction amplification (qRT-PCR) was performed on an ABI 7500 Real Time PCR system (Applied Biosystems; Thermo Fisher Scientific, Inc.). The GAPDH was used as an internal control. The qRT-PCR data was analyzed using the 2−ΔΔCt relative quantitative method. The sequence information of all primers is shown in Table 2.

**Table 2:** Primer sequence list for the three circRNAs for qPCR

Gene	Primer sequence	Annealing temp (°C)	Product size (bp)
GAPDH(HUMAN)	F:5′GGGAAACTGTGGCGTGAT3′ R:5′GAGTGGGTGTCGCTGTTGA3′	60	299
hsa_circRNA_009012	F:5′CCTAGAAGCCACCCAAACTG3′ R:5′CAGGACTTCAGGAAAGCACC3′	60	60
hsa_circRNA_101328	F:5′GGACGGCGTCACCAACCTA3′ R:5′GCACGCATTCTTTCTGGACAT3′	60	150
hsa_circRNA_058230	F:5′TGGATGGGGAGCCCTACAAG3′ R:5′CCAGGTGCGGGTGTACAGG3′	60	94

### Gene ontology and pathway analysis

Among all differentially expressed circRNAs in peripheral blood mononuclear cells of patients with RA, the gene ontology (GO) analysis was performed on the dysregulated target genes of expressed circRNAs, namely, the functional classification of differentially expressed circRNAs. That is, after obtaining GO annotation information, significantly differentially expressed circRNAs were classified into three fields of cellular component (CC), molecular function (MF) and biological process (BP) to further predict the main biological functions that they were involved in. Based on the pathway enrichment analysis database Kobas, the Kyoto Encyclopedia of Genes and Genomes (KEGG) pathway was used for enrichment analysis to explore the potential mechanism of circRNAs involved in the occurrence and development of RA.

### Statistical analysis

Statistical analysis was performed by GraphPad Prism 5.0(version 5.0; GraphPad Software, San Diego, CA, USA) and SPSS 23.0 (version 23.0; SPSS Inc., Chicago, IL, US). The microarray results were achieved by analyzing the obtained images with Agilent feature extraction software (version 11.0.1.1) and processing the data with R software and limma software package. T-tests were applied to compare the measurement data between groups, and the results are presented as x ± s. Spearman’s rank correlation coefficient is used to analyze the correlation between hsa_circRNA_101328 and rheumatoid arthritis (RA), expressed as x ± s. The ROC curve was drawn, and the sensitivity, specificity and area under the curve (AUC) under 95% confidence interval were analyzed. *p*-value less than 0.05 was considered statistically significant.

## Results

### Circular RNA expression profiling in PBMCs from patients with RA

**Fig. 1:**
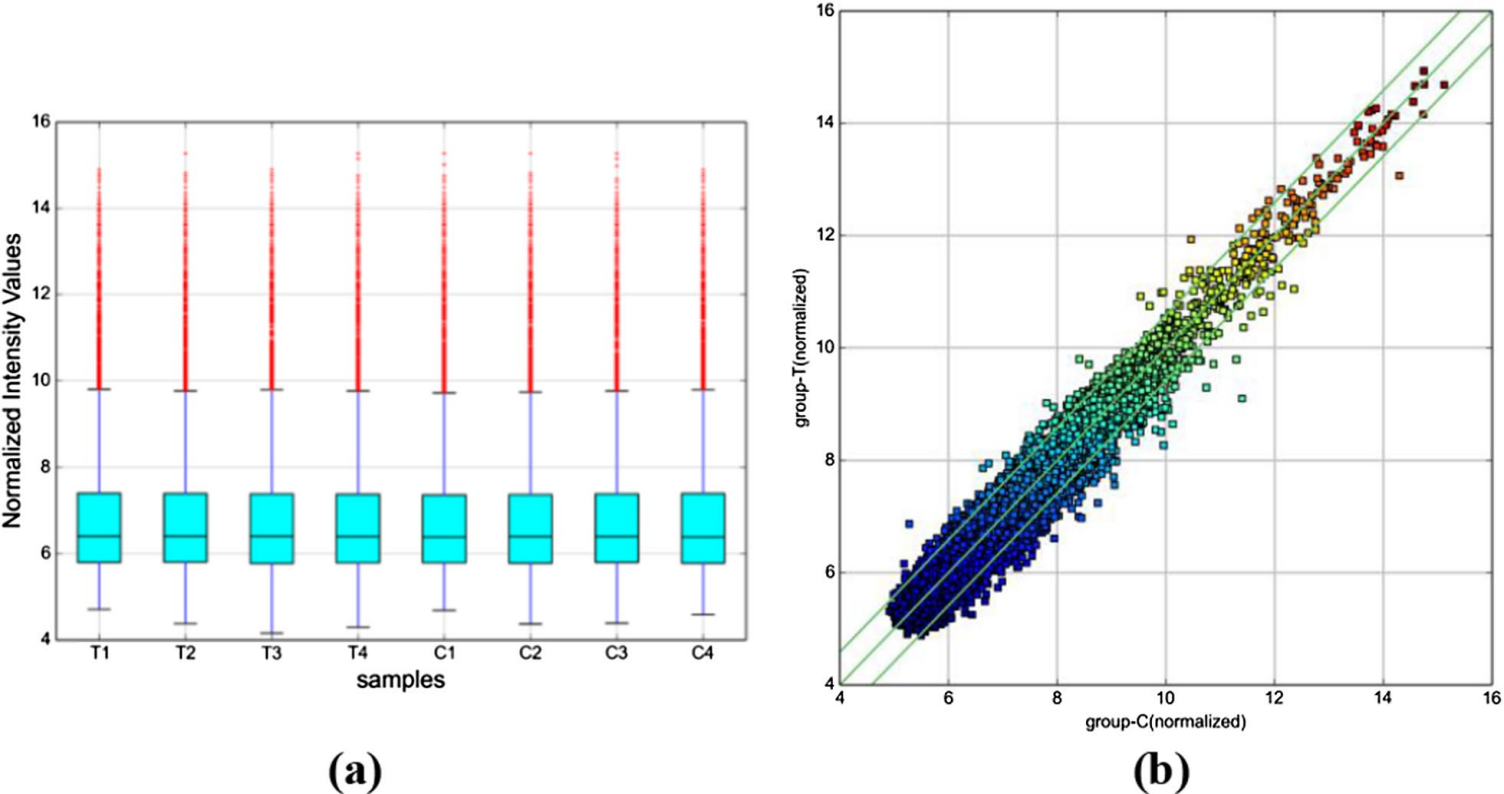
CircRNA expression profile in RA patients, and healthy controls. CircRNA expression was determined by microarray analysis in four RA patients, and four healthy controls. **a** CircRNA Box-Plot. After normalization, the CircRNA data distribution in 8 samples is uniform, and the symmetry is good, and the position of the overall data is approximately on the same horizontal line, and the fluorescent signal strength is approximately consistent, and the next treatment can be performed. **b** CircRNA Scatter-Plot. Parts above the first green line and below the third green line represent circRNA with difference multiple greater than 1.5, and parts between them represent circular RNA with no difference in expression

**Fig. 2:**
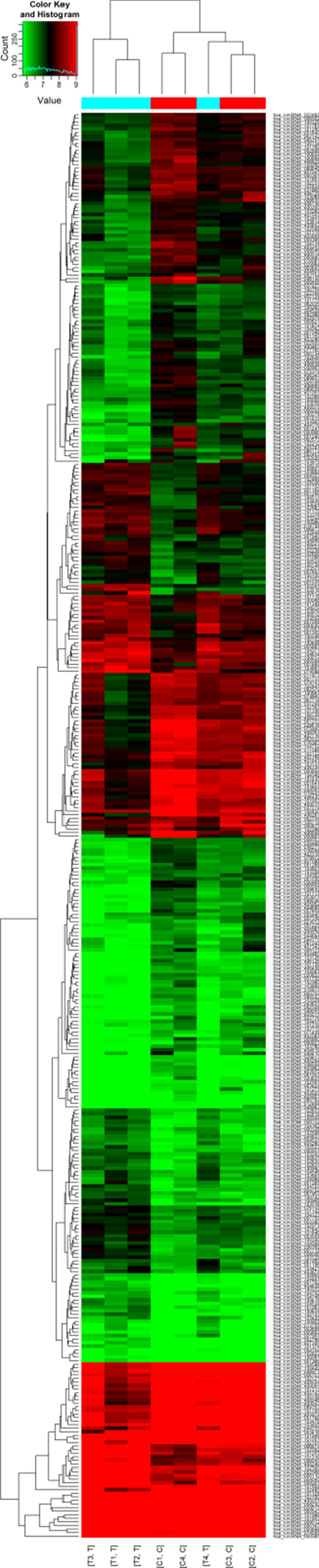
Hierarchical cluster analysis of circRNA. Different expressions of circRNA in different samples can be visually displayed. Each column represents a test sample and each row represents a circRNA. The color shade represented the expression level of circRNA. Red indicated high expression, green indicated low expression, and black indicated no expression. The results show that the difference spectrum is reliable

**Fig. 3:**
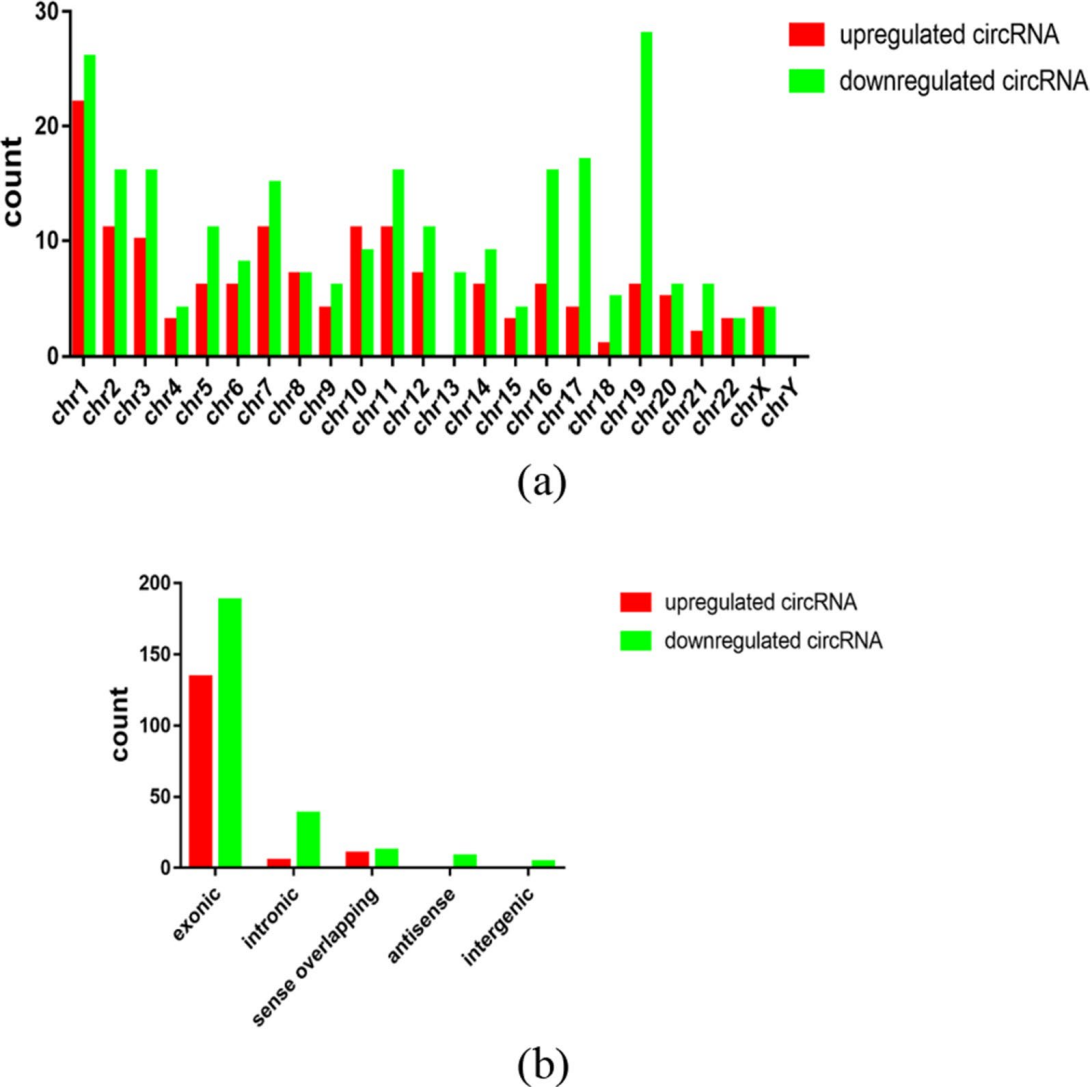
Characteristics of differentially expressed circRNA. **a** Chromosome localization of differentially expressed circRNA. Abbreviation: chr Chromosome. **b** Source region of differentially expressed circRNA

In this study, in order to screen differentially expressed circRNAs, we performed microarray technology on 4 new rheumatoid arthritis patients and 4 sex/age matched healthy controls. A total of 11,088 circRNAs were detected, including 5,802 circRNAs with up expression and 5,286 circRNAs with down expression. The fluorescence signal intensity of the chip can be observed in the box diagram (Fig. 1a) to determine whether the next step should be taken. Scatter graph (Fig. 1b) can directly show the differential expression of circRNAs. In addition, on the basis of all detected circRNAs, a hierarchical clustering analysis of 399 differentially expressed circRNA can more intuitively show the circRNA expression in rheumatoid arthritis and normal samples (Fig. 2). Among these circRNAs, we identified 399 circRNAs that exhibited significant differences (fold change > 1.5, *p* < 0.05) in expression, among which 149 circRNAs were up-regulated and 250 down-regulated. The top 10 abnormally expressed circRNAs were summarized in Table 3 and they were widely distributed in all chromosomes but chromosome Y (Fig. 3a). To be more specific, the top five in distribution were Chrome 1(12.0%, 22/26), Chrome 19(8.0%, 5/28), Chrome 17(7.0%, 11/17) and Chrome2 (7.0%,11/16), Chrome 11(7.0%,12/15). The number of differentially expressed circRNAs originating from exon, intron, sense overlap region, antisense chain and inter-gene was 322(134/188), 43(8/38), 22(10/12), 8(0/8) and 4(0/4), respectively. (Fig. 3b).

**Table 3:** Microarray analysis of circRNAs that are up- or down-regulated in 4 RA patients compared with 4 healthy controls

circRNA	Chrom	circRNA type	Gene symbol	Fold change	*P*	Regulation
hsa_circRNA_009012	chr7	Sense overlapping	STX1A	2.7671084	0.0165	Up
hsa_circRNA_008267	chr3	Exonic	LINC00969	2.6157102	0.0011	Up
hsa_circRNA_074595	chr5	Exonic	ANXA6	2.6082441	0.0287	Up
hsa_circRNA_103516	chr3	Exonic	FNDC3B	2.3412486	0.0435	Up
hsa_circRNA_074598	chr5	Exonic	ANXA6	2.3214408	0.0226	Up
hsa_circRNA_100912	chr11	Exonic	PICALM	2.3109719	0.0416	Up
hsa_circRNA_050898	chr19	Exonic	ACTN4	2.0493023	0.0360	Up
hsa_circRNA_105041	ChrX	Exonic	G6PD	2.0475707	0.0119	Up
hsa_circRNA_001715	chr7	Exonic	LIMK1	1.9267525	0.0093	Up
hsa_circRNA_100969	chr11	Exonic	HINFP	1.8809107	0.0132	Up
hsa_circRNA_102485	chr19	Exonic	PGPEP1	4.9382084	0.0144	Down
hsa_circRNA_044235	chr17	Exonic	CDC27	3.2177705	0.0038	Down
hsa_circRNA_406281	chr3	Intronic	GNL3	2.8108975	0.0250	Down
hsa_circRNA_000881	chr17	Intronic	MSI2	2.7682139	0.0434	Down
hsa_circRNA_102101	chr17	Exonic	CDC27	2.7314357	0.0050	Down
hsa_circRNA_048148	chr19	Exonic	CNN2	2.6229011	0.0399	Down
hsa_circRNA_000858	chr18	Intergenic	–	2.5200677	0.0268	Down
hsa_circRNA_406698	chr5	Intergenic	–	2.5146127	0.0462	Down
hsa_circRNA_000543	chr11	intronic	XLOC_l2_002352	2.4786195	0.0285	Down
hsa_circRNA_001240	chr13	intronic	CUL4A	2.3992491	0.0205	Down

### Interaction between differential expression of circRNAs and miRNAs

Based on TargetScan and miRanda databases, the miRNA target prediction software (Arraystar) was used to predict miRNAs paired with circRNAs and then select top five ones in pairing degree. Studies have shown that circRNAs may be involved in the occurrence and development of diseases through the targeted “sponge” absorption of miRNAs. By consulting literatures, we found that the expression of some miRNAs in our study was correlated with the physiology and pathology of rheumatoid arthritis. The binding sites of the top 10 up-and down-regulated circRNAs and miRNAs in expression are shown in Table 4.

**Table 4:** MiRNA targeted adsorption sites differentially expressing circRNA

CircRNA	MREs
hsa_circRNA_009012	miR-6883-5p, miR-762, miR-6756-5p, miR-4763-3p, miR-6785-5p
hsa_circRNA_008267	miR-4778-3p, miR-6875-3p, miR-4686, miR-7152-5p, miR-200c-5p
hsa_circRNA_074595	miR-4435, miR-3677-5p, miR-3167, miR-4469, miR-4642
hsa_circRNA_103516	miR-147b, miR-19b-1-5p, miR-134-3p, miR-576-5p, miR-493-5p
hsa_circRNA_074598	miR-4435, miR-4642, miR-3677-5p, miR-4691-5p, miR-4685-3p
hsa_circRNA_100912	miR-656-5p, miR-361-3p, miR-9-5p, miR-196a-3p, miR-140-5p
hsa_circRNA_050898	miR-3909, miR-4691-5p, miR-1226-5p, miR-6792-3p, miR-6762-3p
hsa_circRNA_105041	miR-196a-5p, miR-152-5p, let-7b-5p, miR-642a-5p, miR-767-5p
hsa_circRNA_001715	miR-6813-5p, miR-5001-5p, miR-762, miR-4498, miR-324-5p
hsa_circRNA_100969	miR-18b-5p, miR-18a-5p, miR-337-3p, miR-658, miR-149-5p
hsa_circRNA_102485	miR-103a-2-5p, miR-663a, miR-342-3p, miR-188-3p, miR-30b-3p
hsa_circRNA_044235	miR-544a, miR-3127-3p, miR-892a, miR-135b-5p, miR-135a-5p
hsa_circRNA_406281	miR-7843-5p, miR-8063, miR-3670, miR-1250-3p, miR-6879-5p
hsa_circRNA_000881	miR-557, miR-423-3p, miR-323a-5p, miR-541-3p, miR-105-5p
hsa_circRNA_102101	miR-892a, miR-135b-5p, miR-135a-5p, miR-802, miR-338-5p
hsa_circRNA_048148	miR-6775-5p, miR-3187-5p, miR-6089, miR-328-5p, miR-5787
hsa_circRNA_000858	miR-5092, miR-548b-3p, miR-770-5p, miR-374c-3p, miR-6516-5p
hsa_circRNA_406698	miR-3916, miR-6873-5p, miR-4644, miR-5584-5p, miR-1183
hsa_circRNA_000543	miR-338-3p, miR-9-3p, miR-16-2-3p, miR-320b, miR-320a
hsa_circRNA_001240	miR-769-5p, miR-153-3p, 449b-3p, miR-337-3p, miR-602

### Validation of circRNA using RT-qPCR

**Fig. 4:**
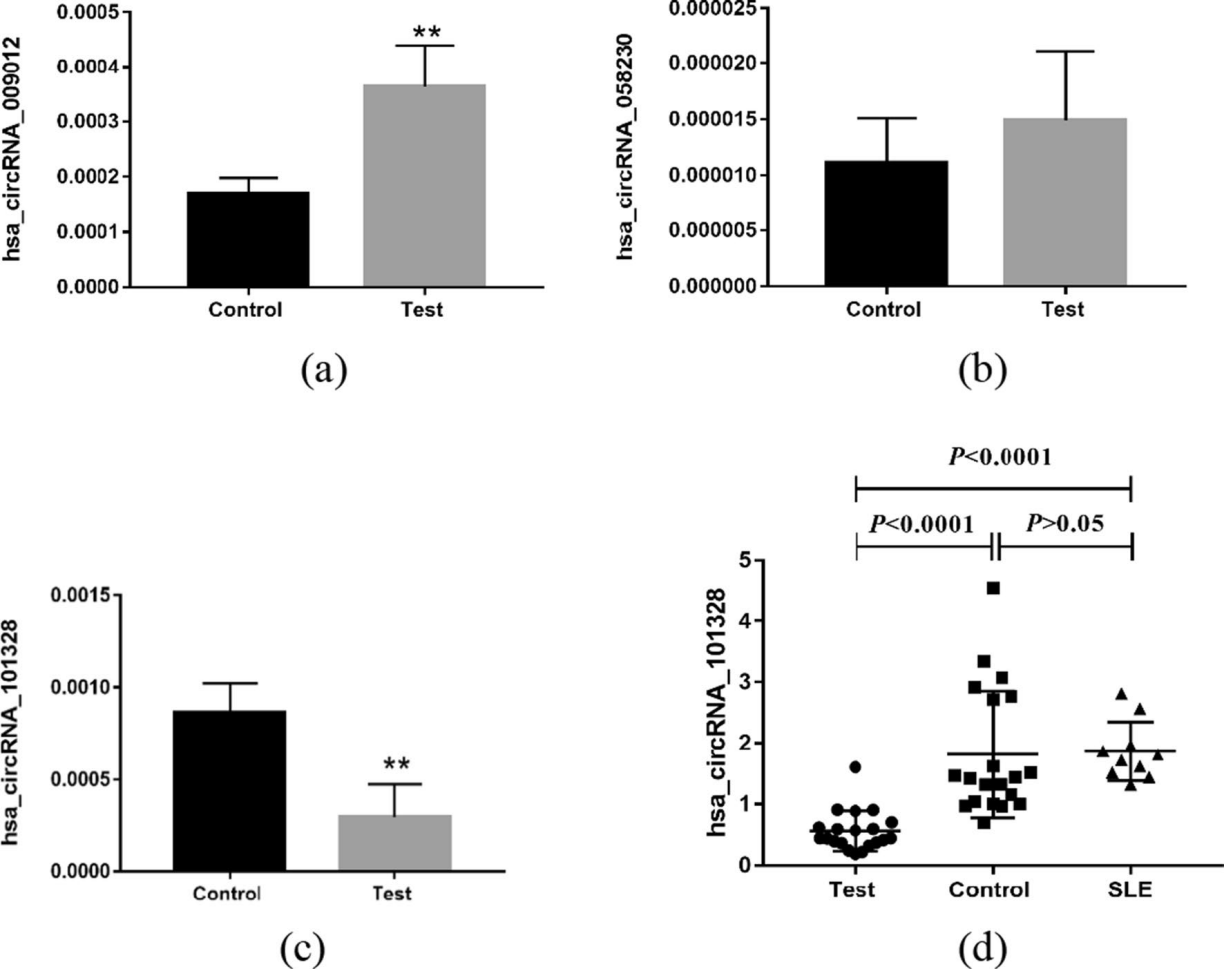
Reverse transcription‑quantitative PCR results of the relative expression levels of circRNAs in PBMCs from patients with RA and the comparison group. **a-c** Hsa_circRNA_009012, hsa_circRNA_050898 and hsa_circRNA_101328 in RA patients were selected for validation. Expression levels of three circRNAs were validated by RT-qPCR in the PBMCs from 4 RA patients, and 4 healthy controls. **d** The expression levels of hsa_circRNA_101328 in PBMCs of 20 RA patients, 10 SLE patients and 20 HC were determined by RT-qPCR. Test: RA patients; Control: healthy controls; SLE: systemic lupus erythematosus patients

To avoid false positives in microarray data, based on the criteria of fold change (FC) > 2.0, *P* < 0.05 and high expression value of original signal,RT-qPCR was used to verify three kinds of circRNAs (hsa_circRNA_058230, hsa_circRNA_009012, hsa_circRNA_101328) among 8 samples used for microarray chip. The results showed that the expression levels of hsa_circRNA_101328 and hsa_circRNA_009012 were significantly different between RA patients with wind-cold-dampness syndrome and healthy controls (*p* < 0.01), whereas there was no significant difference in the expression of hsa_circRNA_058230 (Fig. 4a–c). Then, according to the same criteria, we used RT-qPCR to verify hsa_circRNA_101328 in PBMCs of 20 RA patients, 10 SLE patients and 20 healthy controls. The result revealed that the expression of hsa_circRNA_101328 was significantly downregulated in the PBMCs of RA patients compared to SLE patients and healthy controls. (Fig. 4d).

### Spearman correlation test of clinical variables and confirmed circRNA in PBMCs from RA patients

To determine whether hsa_circRNA_101328, the down-regulated one in expression of RA patients’ PBMCs, has the potential to act as the biomarker related to the severity of rheumatoid arthritis (RA), we conducted Spearman test to further analyze the correlation between the expression of hsa_circRNA_101328 and laboratory indicators of rheumatoid arthritis patients, as shown in Table 5. The expression level of hsa_circRNA_101328 was negatively correlated with c-reactive protein (r = -0.611, *p* < 0.01), but existed no correlation with RF, CCP, ESR, platelet count, lymphocyte count, neutrophil count, leukocyte count, lymphocyte percentage and neutrophil percentage (*p* > 0.01). (Table 5).

**Table 5:** Spearman rank correlation coefficients of hsa_circRNA_101328

Index	hsa_circRNA_101328
hsa_circRNA_101328	1
CRP (mg/l)	− 0.788**
RF (IU/ml)	− 0.091
Anti-CCP (U/ml)	− 0.064
ESR (mm/h)	− 0.177
Thrombocytocrit (%)	− 0.091
Platelet count (10^9^L)	− 0.297
Lymphocyte count (10^9^L)	− 0.216
White cell count (10^9^L)	− 0.318
Neutrophil count (10^9^L)	− 0.206
Lymphocyte percentage (%)	− 0.033
Percentage of neutrophils (%)	− 0.005

CRP, C-reactive protein; RF, rheumatic factor; anti-CCP, anti-cyclic citrullinated peptide antibodies; ESR, erythrocyte sedimentation rate

^*^P < 0.05; **P < 0.01

### ROC curve analysis of circRNAs in PBMCs from RA patients

**Fig. 5:**
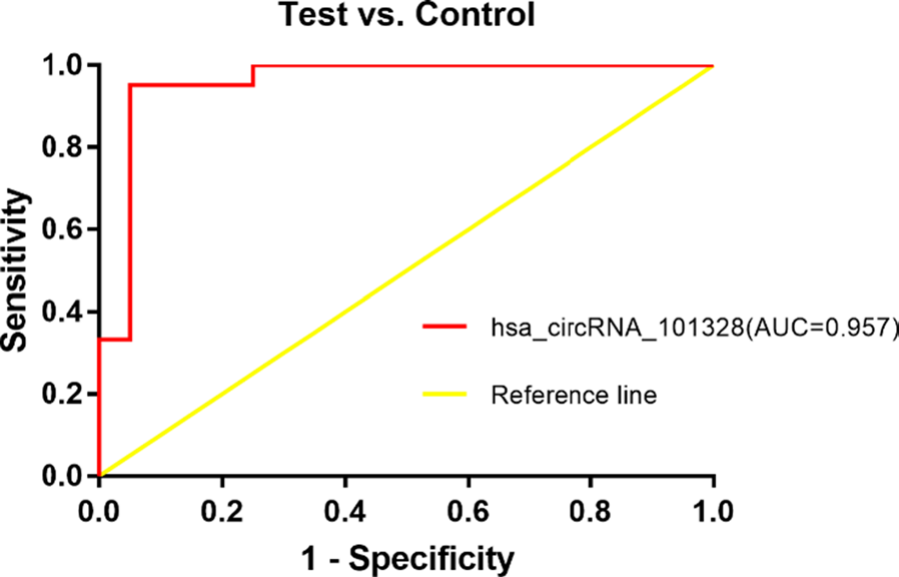
ROC analysis of hsa_circRNA_101328 in PBMCs of RA patients. AUC values are given on the graphs. Test: RA patients; Control: healthy controls

To further evaluate the potential diagnostic value of hsa_circRNA_101328 for rheumatoid arthritis, we used ROC curve and found that the AUC of hsa_circRNA_101328 in peripheral blood mononuclear cells of patients with rheumatoid arthritis was 0.957(95% confidence interval = 0.889–1.025, *p* < 0.01), the sensitivity 95.2%, and the specificity 95%. The cut-off value of hsa_circRNA_101328 was 0.936 (Fig. 5).

### Functional analysis of hsa_circRNA_101328

**Fig. 6:**
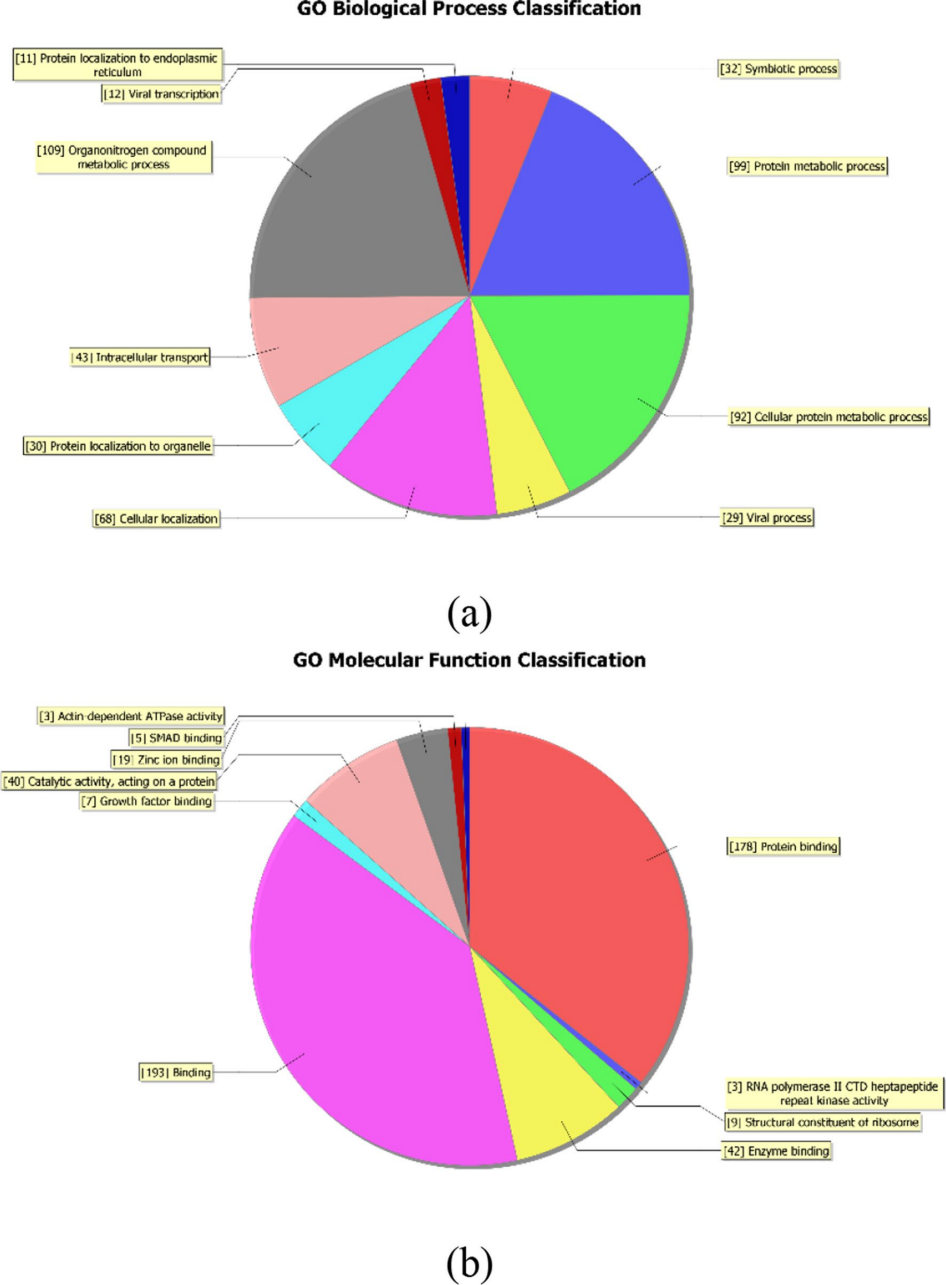

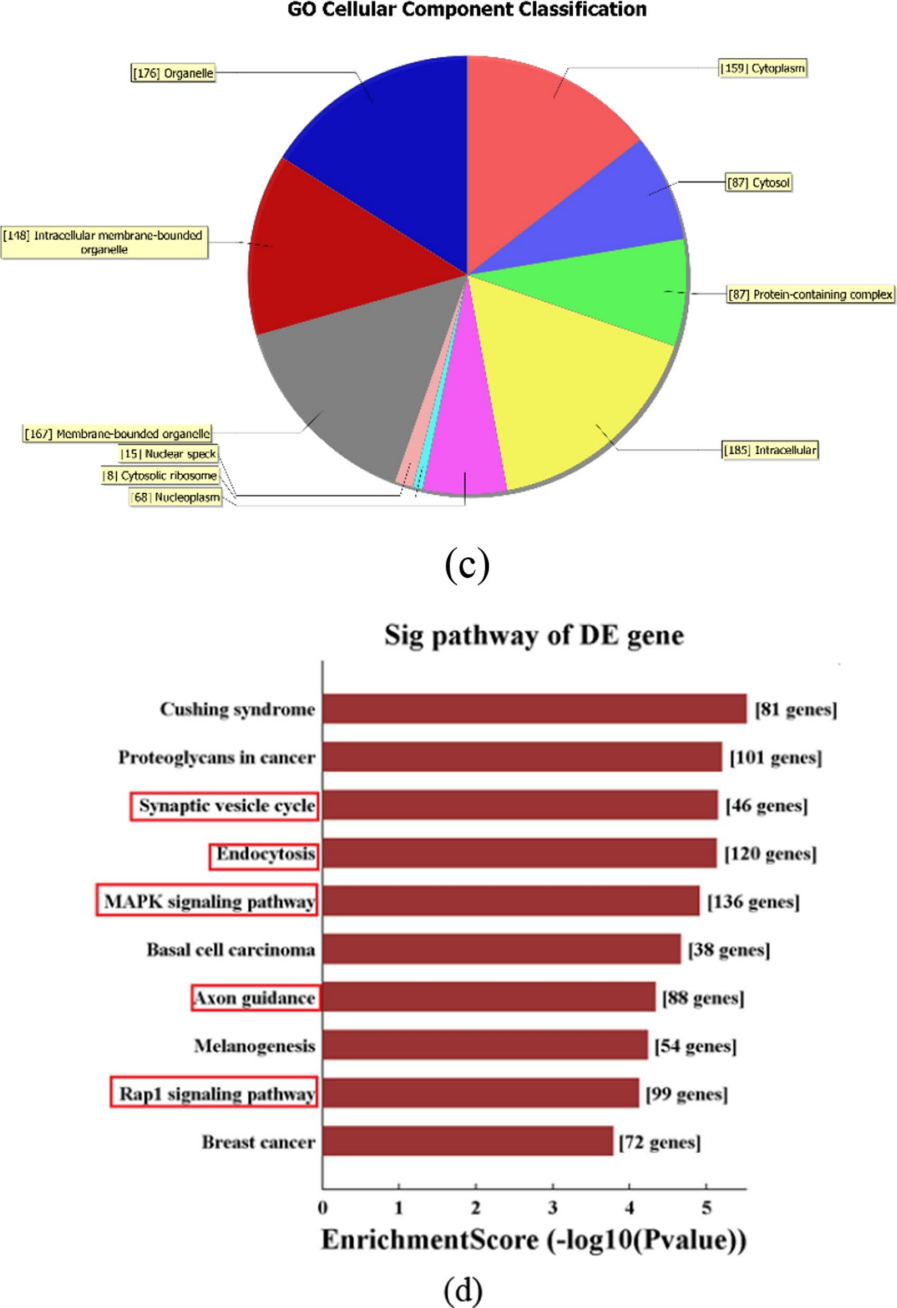
GO and KEGG pathway analyses of hsa_circRNA_101328. **a**–**c** Gene Ontology (GO) analysis for hsa_circRNA_101328-interacting miRNAs and their target genes showing significantly enriched pathways. A indicates biological process (BP), B indicates molecular function (MF), and C indicates cellular component (CC). **d** KEGG Pathway analysis for hsa_circRNA_101328-interacting miRNAs and their target genes. GO: Gene Ontology; KEGG: Kyoto Encyclopedia of Genes and Genomes; Sig pathway: significant pathway

In order to further explore the potential biological functions of hsa_circRNA_101328, GO analysis was performed on the target hsa_circRNA_101328 using gene annotation tool DAVID. The results showed that the gene derived from hsa_circRNA_101328 was manifested in protein binding, membrane binding and transcriptional regulation activity in biological process. In terms of cell components, it was manifested in cell connection, intimal system, cytoplasm etc.; In the aspect of molecular function, it was manifested in the positive regulation of cell process and the development of anatomical structure (Fig. 6a–c). The KEGG pathway analysis of hsa_circRNA_101328 was carried out using KOBAS database, and the results found that hsa_circRNA_101328 may be involved in synaptic vesicle circulation, endocytosis, MAPK signaling pathway, axon orientation and Rap1 signaling pathway, and then the occurrence and development of RA [12] (Fig. 6d).

## Discussion

RA is one of the most common autoimmune diseases, and it occurs most frequently in the elderly, especially among female [1]. The main pathological manifestations are significant proliferation of synovial cells, diffuse inflammatory cytokines and tumor-like invasive granulation tissue promoting cartilage erosion and bone destruction, and eventually the irreversibility of joint deformities and functional loss at the focus [13], which to a great extent reduces patients’ life quality. Therefore, rheumatoid arthritis of great importance, and the method of early diagnosis need to be explored earlier.

CircRNAs, a new type of non-coding single-stranded RNA with closed structure, was first found in plant viruses in 1976, and later found widespread in eukaryotes [14, 15]. The special closed-loop structure of circRNA makes it difficult to be recognized by exonucleautomotive service engineers, and it can remain stable and conserved the evolution process [16]. Meanwhile, more and more studies proved that it could stably exist in whole blood, blood cells and extracellular vesicles [17]. At present, the biological functions of circRNA have been continuously explored, such as circRNA’s “sponge” absorption of miRNA, competitive regulation of downstream target genes [18], as well as the regulation of gene transcription and RNA-binding protein, direct translation and synthesis of protein, etc. As a long-chain non-coding RNA, circRNA is extensively involved in the regulation of human activities and related diseases. The special structure, features and biological function of circRNA determine that it can serve as a potential biomarker of diseases. To date, a host of domestic and foreign literature reports have confirmed that circRNA is widely involved in the occurrence and development of diseases. Such as, abnormal expression of circRNA in cardiovascular diseases, various cancers, nervous system diseases and other diseases, and also involved in the pathogenesis of diseases and reflects the severity of diseases [19–21]. Additionally, the research on its relationship with autoimmune diseases remains a public focus, for example, circSnx5 has been confirmed to be a potential therapeutic pathway for immune-related diseases [22]. Also, it was confirmed that circRNAs could be involved in the inflammatory response of RA through acting on RA inflammatory cytokines. For example, hsa_circ_0003353 [23] was confirmed to have significantly higher expression in RA -FLS. Meanwhile, after the inhibition and over-expression of hsa_circ_0003353, the proliferation, migration, invasion, apoptosis of RA-FLS and the production of inflammatory cytokines showed significant changes, suggesting that hsa_circ_0003353 may play an important role in the promotion of immunity, inflammation, synovial infiltration and joint destruction. After the down-regulation of circ_AFF2 [24], the cell cycle, proliferation and inflammatory response of RA-FLS were blocked, while apoptosis was promoted. In addition, circ_AFF2 can induce cellular processes and inflammatory responses in FLS-RA cells through the miR-375/TAB2 axis. Hsa_circ_0088036 [25] promotes synovial disease by acting as a sponge for miR-140-3p to upregulate SIRT 1 expression. At the same time, hsa_circ_0044235 [26] and circRNA_104871 [27] have been confirmed to act as novel biomarkers for RA.

In this study, we identified 11,088 differentially expressed circRNAs in RBMCs of RA patients and healthy controls using the microarray technology, among which 5802 were up-regulated and 5286 down-regulated. According to the FC and P values, the total number of differentially expressed circRNA in PBMCs of patients with RA was 399, including 149 up-regulated circRNAs and 250 down-regulated ones. Prediction of miRNA binding sites and related signaling pathways of differentially expressed circRNA were studied, and literature review of miRNA binding sites revealed that MiR-338-5p promotes the proliferation, invasion and inflammatory response of fibroblast-like synoviocytes in RA by regulating SPRY1 [28]. MiR-9-5p [29] and miR-6089 [30] have anti-inflammatory and cartilage protective effects, and they are important mediators of the pathogenesis of RA. MiR-135b-5p [31] was associated with increased disease risk and activity of RA, while hsa_circRNA_100912, hsa_circRNA_102101, hsa_circRNA_102485, hsa_circRNA_048148, and hsa_circRNA_044235 had targeted adsorption sites with the above-mentioned miRNAs, It suggested that these circRNAs might participate in the occurrence and development of RA through targeted sponge absorption of miR-338-5p, miR-9-5p, miR-6089, mi R-342-3p, and miR-135b-5p; Many researchers have explored new genes or transcription factors involved in the biological functions of diseases in Parkinson's disease, diabetes, rheumatoid arthritis and other diseases based on network biology methods, providing new ideas for the occurrence and development of diseases [32–34]. In this study, GO analysis found that hsa_circRNA_101328-derived genes exhibited protein binding, membrane binding and transcriptional regulatory activities in biological processes. In terms of cell components, it was manifested in cell connection, intimal system, cytoplasm etc.; In the aspect of molecular function, it was manifested in the positive regulation of cell process and the development of anatomical structure. KEGG pathway analysis found that hsa_circRNA_101328 may be involved in synaptic vesicle circulation, endocytosis, MAPK signaling pathway, axon orientation and Rap1 signaling pathway, and then the occurrence and development of RA.

In addition, false positives may occur in microarray results. To test the accuracy of microarray results, three differentially expressed circRNAs (hsa_circRNA_101328, hsa_circRNA_009012, hsa_circRNA_050898) screened by microarray chips were selected in accordance with relevant requirements in this study. RT-qPCR technique was used to verify the expression levels of hsa_circRNA_101328, hsa_circRNA_009012 and hsa_circRNA_050898 in the peripheral blood of 4 RA patients with wind-cold-dampness obstruction pattern and 4 healthy subjects. The result found that the expression levels of hsa_circRNA_101328 and hsa_circRNA_009012 were significantly different between RA patients with wind-cold-dampness obstruction pattern and healthy objects. While the expression level of hsa_circRNA_050898 was not significantly different between RA patients with wind-cold-dampness obstruction pattern and healthy objects. The expanded sample size of hsa_circRNA_101328, which were consistent with microarray results, were selected for qRT-PCR verification. The present study demonstrated significant differences in hsa_circRNA_101328 expression between RA patients, SLE patients and HCs in three validation cohorts. Although the expression of hsa_circRNA_101328 was significantly downregulated in the PBMCs of RA patients compared to SLE patients and healthy controls., suggesting that hsa_circRNA_101328 could be a common diagnostic circRNAs for RA. The lack of difference in the expression of hsa_circRNA_050898 is related to individual differences between patients, such as disease severity, disease duration, and the presence of complications, which may affect the accuracy of verification results. Also, the correlation between the expression of hsa_circRNA_101328 and RA clinical laboratory indexes was analyzed, and the results showed that hsa_circRNA_101328 had a significantly negative correlation with CRP and no correlation with RF, CCP, ESR, platelet aggregation, platelet count, lymphocyte count, neutrophil count, leukocyte count, lymphocyte percentage and central granulocyte percentage. The results showed that: The disease activity of RA is closely related to CRP and ESR, and this study suggests that hsa_circRNA_101328 reflects the disease activity of RA. ROC curve analysis of hsa_circRNA_101328 revealed that the area under the curve was 0.957, suggesting that hsa_circRNA_101328 could be used as a molecular marker for the diagnosis of wind-cold-dampness obstruction pattern in RA. However, research on diagnostic markers has some limitations. First, the sample size is not big enough, and the sample size can be expanded later, and it can be confirmed in people from different regions and different ethnic groups. Second, in order to prove that hsa_circRNA_101328 can be used as a diagnostic marker of rheumatoid arthritis, it should be proved that it can effectively distinguish rheumatoid arthritis from other rheumatic diseases, such as systemic lupus erythematosus and ankylosing spondylitis. Further studies will be carried out, adding the argument of hsa_circRNA_101328 as a diagnostic marker for rheumatoid arthritis.

## Conclusions

In conclusion, we screened the differentially expressed circRNAs in PBMCs of RA patients using microarray technology, and performed RT-qPCR and ROC curve analysis on them, confirmed that hsa_circRNA_101328 is expected to become a potential molecular marker for the diagnosis of RA. Its regulation may be involved in the occurrence and development of RA through the regulation of relevant signaling pathways by target gene miRNA, but the specific mechanism needs further investigation.

## Data Availability

The datasets generated and/or analysed during the current study are available in the GEO repository, the GEO accession number is GSE189338, available at: https://www.ncbi.nlm.nih.gov/geo/query/acc.cgi?acc=GSE189338.

## Abbreviations

RA: Rheumatoid arthritis
CircRNAs: Circular RNAs
PBMCs: Peripheral blood mononuclear cells
RT-qPCR: Reverse transcription-quantitative polymerase chain reaction
GO: Gene ontology
KEGG: Kyoto encyclopedia of genes and genomes
CRP: C-reactive protein
RF: Rheumatic factor
anti-CCP: Anti-cyclic citrullinated peptide antibodies
ESR: Erythrocyte sedimentation rate
SLE: Systemic lupus erythematosus
